# Refining surgical strategies in ThuLEP for BPH: a propensity score matched comparison of En-bloc, three lobes, and two lobes techniques

**DOI:** 10.1007/s00345-024-05136-5

**Published:** 2024-07-22

**Authors:** Francesco Cantiello, Fabio Crocerossa, Stefano Alba, Umberto Carbonara, Savio Domenico Pandolfo, Ugo Falagario, Alessandro Veccia, Giuseppe Ucciero, Matteo Ferro, Nicola Mondaini, Rocco Damiano

**Affiliations:** 1 Department of Urology, Magna Graecia University of Catanzaro, Viale Europa, 100 Catanzaro (CZ), Catanzaro, 88100 Italy; 2 Department of Urology, Romolo Hospital, Rocca di Neto (Kr), Crotone, Italy; 3 Unit of Urology, GVM - Santa Maria Hospital, Bari, Italy; 4 Department of Emergency and Organ Transplantation-Urology, University of Bari, Bari, Italy; 5 Department of Urology, University of L’Aquila, L’Aquila, Italy; 6 Department of Urology and Kidney Transplantation, University of Foggia, Foggia, Italy; 7 Department of Molecular Medicine and Surgery, Karolinska Institutet, Stockholm, Sweden; 8 Department of Urology, University of Verona, Verona, Italy; 9 Unit of Urology, R. Dulbecco Hospital, Catanzaro, Italy; 10 Division of Urology, European Institute of Oncology (IEO)-IRCCS, Milan, Italy

**Keywords:** Laser enucleation, En-bloc, Two-lobe, Three-lobe, BPH, BPO

## Abstract

**Purpose:**

This study compares the peri-operative and functional outcomes of three distinct surgical techniques in Thulium Laser Enucleation of the Prostate (ThuLEP) for benign prostatic hyperplasia (BPH). The main aim is to assess whether the En-bloc, Three-lobe, and Two-lobe techniques have differential effects on surgical efficacy and patient outcomes.

**Methods:**

A retrospective analysis was conducted on patients undergoing ThuLEP for BPH between January 2019 and January 2024 at two tertiary centers. Propensity score matching was utilized to balance baseline characteristics among patients undergoing the different techniques. Surgical parameters, including operative time, enucleation time, morcellation time, energy consumption, and postoperative outcomes, were compared among the groups.

**Results:**

Following propensity score matching, 213 patients were included in the analysis. Intraoperative analysis revealed significantly shorter enucleation, laser enucleation, morcellation and operative times and total energy delivered in the En-bloc and Two-lobe groups compared to the Three-lobe group. No significant differences were observed among the groups in terms of intraoperative and postoperative complications. There were no significant differences in functional outcomes at the 3-month follow-up among the groups.

**Conclusion:**

The findings of this study suggest that while the En-bloc and Two-lobe techniques may offer efficiency benefits and could be considered safe alternatives in ThuLEP procedures, the reduction in laser enucleation time and energy delivered did not necessarily translate into improvements in post operative storage symptoms or other functional outcomes for the patients. Surgeon preference and proficiency may play a crucial role in selecting the most suitable technique for individual patients. Future research should focus on larger-scale prospective studies to further validate these findings and explore potential factors influencing surgical outcomes.

**Supplementary Information:**

The online version contains supplementary material available at 10.1007/s00345-024-05136-5.

## Introduction

Endoscopic enucleation of the prostate (EEP) is a well-established treatment for lower urinary tract symptoms (LUTS) due to benign prostatic obstruction (BPO)/benign prostatic hyperplasia (BPH), regardless of prostate volume [1]. Laser EEP offers effective obstruction relief with improved safety compared to trans-urethral resection of the prostate (TURP) and open simple prostatectomy (OP) [2, 3].

The American Urological Association (AUA) guidelines recommend laser EEP for the treatment of BPO ≥ 80 ml [4], whereas the European Association of Urology (EAU) guidelines recommend laser or bipolar EEP as a valid alternative treatment respect of TURP in the treatment of prostate volumes ranging between 30 and 80 ml, and laser or bipolar EEP together with open prostatectomy as standard of care in the treatment of prostates with a volume > 80 ml [5].

Since HoLEP’s introduction [6], other techniques emerged. Plasmakinetic bipolar enucleation of the prostate (BipoLEP) showed similar efficacy to HoLEP but with less morbidity [2, 3, 7]. Subsequently, Thulium laser vapo-enucleation (ThuVEP), and Thulium Laser Enucleation (ThuLEP), were developed [8, 9]. ThuLEP is as effective as HoLEP in relieving LUTS [10, 11], with lower morbidity compared to TURP or OP [2, 3].

The convergence of outcomes observed in HoLEP, ThuLEP, and other EEP methodologies (such as Green light laser, and BipoLEP), influenced a shift in the scientific debate from determining the optimal energy source to identifying the most effective surgical approach. Laser EEP typically entails identifying the surgical capsule followed by retrograde enucleation; in the Three-lobe technique [10], both side lobes and the middle lobe of the prostate are enucleated separately, while in the Two-lobe and En-bloc technique, the middle lobe is enucleated after the side lobes or concurrently with them [12, 13].

Several studies on HoLEP compared the three enucleation techniques in terms of intra- and peri-operative parameters and outcomes [14–16]. However, there are no studies available investigating potential differences for ThuLEP. The aim of this study was to compare the peri-operative and functional outcomes of the Three-lobe, Two-lobe, and En-bloc techniques in ThuLEP.

## Materials and methods

### Patient population

A retrospectively maintained database containing data of patients with LUTS secondary to BPH undergoing ThuLEP in two tertiary centers over the last 5 years was used. Exclusion criteria comprised prior prostate surgery, prostate cancer, urethral strictures, bladder neck sclerosis, bladder stones, overactive bladder and urinary tract infections. Inclusion criteria consisted of a prostate volume > 60 ml, failure or non-compliance to medical treatment, International Prostate Symptom Score (IPSS) ≥ 8, maximal urine flow rate (Qmax) less than 15 ml/sec, post-void residual (PVR) ≥ 50 ml, and/or all absolute indications for surgery according to EAU guidelines [5].

Surgery was performed by two urologists with equal levels of expertise (> 50 procedures). Antiplatelet drugs were discontinued 7 days prior to surgery, while oral anticoagulants were replaced by low-molecular-weight heparin, which was stopped 12 h before surgery. Preoperatively, age, IPSS (International Prostate Symptom Score), IPSS-QoL (IPSS Quality of Life), Qmax (maximum urinary flow rate), PVR (post-void residual volume), prostate volume, PSA (prostate-specific antigen), Hb (hemoglobin) levels, ASA (American Society of Anesthesiologists) score, presence of indwelling catheter, and 5-ARI (5-alpha-reductase inhibitor) intake were evaluated. Surgical parameters encompassed operative time (OT), adenoma weight, enucleation time (ET), morcellation time (MT), laser enucleation time (LET), and energy consumption per procedure. These metrics were also standardized by expressing them as rates per gram of adenoma to mitigate the influence of variations in adenoma size across procedures. Length of hospital stay (LOS), catheterization time (days) were collected. Intraoperative and postoperative complications at 3 months were reported according to the modified Clavien-Dindo classification [17]. All patients were evaluated at the 3-month postoperative mark for functional parameters. A 24-hour pad test was used to evaluate the prevalence of stress and urge incontinence. The pad test was considered positive if there was an increase in pad weight exceeding a predetermined threshold of 2 g. To distinguish between stress and urge incontinence, the pad test was combined with patient diaries detailing the circumstances of leakage. All patients provided informed consent for data collection and case presentation.

### Surgical procedure

All ThuLEP procedures were performed using a continuous flow 26 French resectoscope equipped with a 12° Hopkins optic. A continuous wave 200 Watts Thulium: YAG laser (Quanta System) was utilized, with power adjusted to 70 Watts for cutting (2.4 Joules and 30 Hz) and 30 Watts for coagulation (2 Joules and 15 Hz) throughout the procedure. A 550-micron optical core, bare end, reusable laser fiber was employed. Enucleation commenced at the prostatic apex to perform an early apical urethral mucosa release. Early apical release was applied to all enucleation techniques in order to eliminate its influence on the evaluated outcomes. An inverted U-shaped incision was made around the verumontanum, extending 1 cm upwards, until the surgical plane was identified. A marking incision was then made at 12 o’clock at the apex, and connected circumferentially with the end of the inverted U-shaped incision lines.

In the En-bloc technique, an initial incision was made from the bladder neck to the verumontanum at 5 o’clock until the surgical plane was identified. Enucleation of the left lobe commenced at the apex towards the bladder neck with an ascending incision from 5 to 11 o’clock counterclockwise. The same procedure was performed in the opposite direction for the right lobe, with the median lobe enucleated together with it.

The Two-lobe technique involved an initial incision from the bladder neck to the verumontanum at 5 o’clock until the surgical plane was identified. A deep incision was then made at 12 o’clock, followed by enucleation of the left lobe from the apex towards the bladder neck with an ascending incision from 5 to 12 o’clock counterclockwise. The same procedure was repeated for the median and right lobe.

The Three-lobe technique entailed the detachment of the third lobe and each side lobe independently. A deep incision was made at the 12 o’clock position to perform an anterior commissurotomy. The median lobe was enucleated first, with a double incision from the bladder neck to the apex at 5 and 7 o’clock, followed by a retrograde ascending enucleation. Dissection of the lateral lobes commenced with a retrograde ascending incision from 5 to 12 o’clock counterclockwise on the left, and from 7 to 12 o’clock clockwise on the right. Morcellation was performed using a Piranha morcellator. A 22-French three-way catheter was inserted for continuous bladder irrigation with normal saline.

### Statistical analysis

Baseline, intraoperative, and postoperative variables were collected. Means and standard deviations were reported for continuously coded variables, while categorical variables were reported using frequencies and proportions.

To mitigate potential confounding effects and balance baseline characteristics between treatment groups, a propensity score was calculated for each patient based on age, baseline IPSS, IPSS-QoL, Qmax, PVR, prostate volume, PSA, Hb levels, ASA score, presence of indwelling catheter, and 5-ARI intake. The propensity score was then used to match patients across treatment groups in a 1:1:1 ratio for En-bloc, Two-lobe, and Three-lobe techniques. The En-bloc patient cohort was used for pairwise matching the other two groups.

Balance between cohorts was assessed using ANOVA and Chi-squared tests. Intraoperative and postoperative outcomes were compared using the same statistical approach. Specifically, Fisher’s ANOVA test was used to compare continuous preoperative, intraoperative, and postoperative variables between the treatment groups. The Levene test was used to test the assumption of equality of variance between groups. In case of significant differences in Fisher’s ANOVA, Tukey’s post-hoc multiple comparison test was applied to further identify significant pairwise differences. In case of a violation of homoscedasticity, differences between groups were assessed using Welch’s ANOVA analysis, while Tamhane’s T2 was performed as a post-hoc test. Differences in proportions for nominal variables were assessed using the Chi-square test, and differences between ordinal data variables were assessed using the non-parametric Kruskal-Wallis test.

All tests were two-sided, with statistical significance set at *p* < 0.05. Analyses were conducted using SPSS Statistics, version 27 (IBM, Armonk, NY, USA), and GraphPad Prism 9 (GraphPad Software, Inc., San Diego, CA).

## Results

331 patients met the inclusion criteria and underwent subsequent propensity score calculation. Following pairwise propensity matching, 71 patients for each enucleation technique were selected from the database, totaling 213 cases. No significant differences in any preoperative baseline characteristics were observed among the cohorts (Supplementary Table 1). The Levene test results are provided in Supplementary Table 2.

ANOVA analysis of intraoperative outcomes revealed significant differences among treatment cohorts in terms of ET, ET per gram, LET, OT, OT per gram (*p* < 0.001 for all) and energy applied (*p* = 0.013). However, no statistically significant differences were noted in enucleated adenoma weight (*p* = 0.079), LET per gram (*p* = 0.6), energy per gram (*p* = 0.3), or the incidence of intraoperative complications (*p* = 0.17) (Table 1).

**Table 1:** Intraoperative outcomes stratified by enucleation technique (En-bloc (*n* = 71), two-lobe (*n* = 71), three-lobe (*n* = 71)

Variable	Group	Mean (SD)	Anova F	*p*
Enucleated adenoma weight (g)	En-bloc	71 (102.8)	2.882*	0.079*
	Two-lobe	71 (99.2)		
	Three-lobe	71 (115.8)		
ET per gram (min/g)	En-bloc	0.7 (0.2)	8.461	***< 0.001***
	Two-lobe	0.7 (0.2)		
	Three-lobe	0.8 (0.2)		
ET (min)	En-bloc	71.6 (19.7)	31.184*	< 0.001*
	Two-lobe	67.5 (13.8)		
	Three-lobe	90.3 (20.2)		
MT per gram (min/g)	En-bloc	0.2 (0.1)	25.185*	< 0.001*
	Two-lobe	0.2 (0.2)		
	Three-lobe	0.3 (0.1)		
MT (min)	En-bloc	19.3 (12.7)	16.849*	< 0.001*
	Two-lobe	20.5 (20.1)		
	Three-lobe	35.7 (20.8)		
LET per gram (min/g)	En-bloc	0.5 (0.2)	0.438*	0.65*
	Two-lobe	0.5 (0.2)		
	Three-lobe	0.6 (0.2)		
LET (min)	En-bloc	49.4 (8.7)	19.672	***< 0.001***
	Two-lobe	48.1 (9.5)		
	Three-lobe	56.5 (7.2)		
Energy per gram (KJ/g)	En-bloc	1.5 (0.8)	1.270	*0.28*
	Two-lobe	1.7 (0.7)		
	Three-lobe	1.6 (0.8)		
Energy (KJ)	En-bloc	136.6 (51.9)	4.526*	0.013*
	Two-lobe	147.7 (30.3)		
	Three-lobe	163.1 (53.3)		
OT per gram (min/g)	En-bloc	0.9 (0.2)	27.66*	< 0.001*
	Two-lobe	0.9 (0.3)		
	Three-lobe	1.1 (0.2)		
OT (min)	En-bloc	91 (29.2)	25.826	***< 0.001***
	Two-lobe	88 (27.9)		
	Three-lobe	125.9 (37.8)		
Intraoperative complications		**NO (n**,**%)**	**YES (n**,**%)**	
	En-bloc	71 (100%)	0 (0%)	0.17**
	Two-lobe	70 (98.6%)	1 (1.4%)	
	Three-lobe	68 (95.6%)	3 (4.4%)	

SD, standard deviation; ET, enucleation time; MT, morcellation time; LET, laser enucleation time; OT, operative time; * Welch test; ** Chi- squared test

**Fig. 1:**
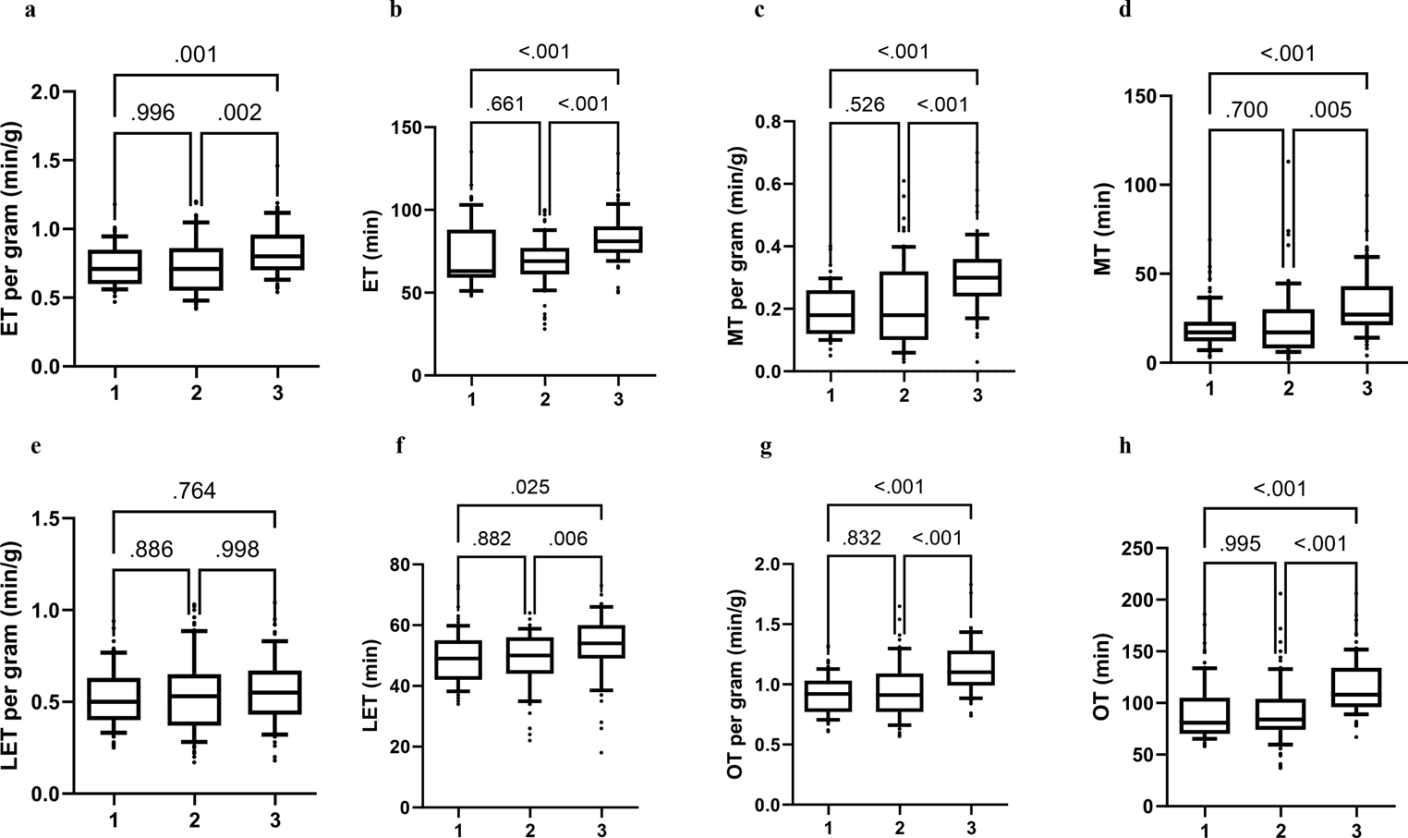
Multiple comparison test of intraoperative outcomes stratified by enucleation technique (En-bloc (*n* = 71), Two-lobe (*n* = 71), Three-lobe (*n* = 71). ET, enucleation time; MT, morcellation time; LET, laser enucleation time; OT, operative time; The Tamhane’s t2 test was applied for MT, MT per gram and OT comparison. The p-value for each pairwise comparison is depicted above the respective bracket

Multiple comparison tests for intraoperative variables indicated a significantly shorter ET per gram in the En-bloc compared to the Three-lobe technique, with a mean difference (MD) of -0.11 min/g (*p* = 0.001) in favor of the former. Similarly, the Two-lobe technique exhibited a mean ET per gram of -0.11 min/g lower than the Three-lobe technique (*p* = 0.002). However, no significant differences were found in ET per gram between the En-bloc and Two-lobe techniques (*p* = 0.99). ET was significantly shorter for both En-bloc and Two-lobe techniques compared to the Three-lobe technique (MD -18.7 min, *p* < 0.001 and MD -22.8 min, *p* < 0.001, respectively), with no differences between En-bloc and Two-lobe techniques (*p* = 0.7). Significant differences in LET were observed between the En-bloc and Three-lobe techniques (MD = -7.1 min, *p* = 0.025) and between the Two-lobe and Three-lobe techniques (MD = -8.3 min, *p* = 0.006), with no differences between En-bloc and Two-lobe techniques (*p* = 0.9). However, no significant differences were observed among the three techniques in LET per gram (*p* = 0.6). Even though the ANOVA for the absolute energy delivered showed significant differences among groups, the subsequent post-hoc test did not unveil any significant differences across the three comparisons (En-bloc vs. Two-lobe *p* = 0.2; En-bloc vs. Three-lobe *p* = 0.085; Two-lobe vs. Three-lobe *p* = 0.071) (Supplementary Fig. 1). Both En-bloc and Two-lobe techniques exhibited significantly shorter OT per gram compared to the Three-lobe technique (MD = -0.23 min/g, *p* < 0.001 and MD = -0.22 min/g, *p* < 0.001, respectively), as well as shorter OT (MD = -34.9 min, *p* < 0.001 and MD = -37.9 min, *p* < 0.001, respectively). No significant differences were found between En-bloc and Two-lobe techniques in either OT per gram (*p* = 0.9) or OT (*p* = 0.8) (Fig. 1, Supplementary Table 3).

No significant differences were noted among the three surgical groups in terms of catheterization time, LOS, postoperative Hb loss, or rates of postoperative overall, minor and major complications. Additionally, no significant differences were observed in functional outcomes at the 3-month follow-up, including changes in Qmax, total IPSS, IPSS-QoL, PVR, and PSA levels. Similarly, no differences were observed in the rates of stress incontinence and urge incontinence at 3 months among the groups (Supplementary Table 4).

## Discussion

In recent years, three distinct surgical approaches to laser EEP have emerged, all designed to emulate adenoma enucleation along the surgical capsule in open prostatectomy [18]. Initially introduced for holmium laser, these approaches include the Three-lobe technique [6]; the Two-lobe technique, introduced to simplify the procedure and facilitate the learning curve [19]; and the En-bloc technique proposed by Scoffone to streamline surgical time by identifying the surgical plane only once [20].

Comparative studies between these HoLEP techniques are limited, showing no clinically significant differences in perioperative outcomes (Hb drop, catheterization time, LOS) and functional outcomes. However, both the En-bloc and Two-lobe techniques demonstrate significantly shorter OT compared to the Three-lobe technique [14–16].

ThuLEP has proven to be safe and effective, comparable to HoLEP for prostates of all sizes, with no apparent difference in clinical outcomes between the two laser types [10, 21]. Additionally, some studies suggest a shorter learning curve and reduced operative time for ThuLEP, attributed to the intrinsic characteristics of the thulium laser [22, 23].

Herrmann et al. introduced the Three-lobe technique for ThuLEP in 2010 [9]. Subsequent modifications included the all-in-one lobe ThuLEP technique introduced by Kim et al. [12], and two distinct En-bloc procedures presented by Castellani et al. [24]. These techniques offer enhanced control of the surgical capsule, reduced OT and improved visual clarity due to decreased incidence of hematuria.

These advantages have been confirmed by Enikeev et al. [13], who showed that both En-bloc and Two-lobe ThuLEP were comparable in terms of peri- and post-operative outcomes and complications, suggesting that the choice of technique should be based on surgeon preference rather than surgical approach. Saredi et al. [25] also confirmed the benefits of En-bloc ThuLEP in terms of operative time and energy delivered per gram of adenoma in a comparative study with the traditional Three-lobe technique.

In our study, we found a significant reduction in total enucleation and enucleation per gram in both the En-bloc and Two-lobe technique groups. This can be attributed to the need to identify the surgical plane only once or twice, compared to three times in the standard technique, reducing the risk of mismatching incisions and subsequent complications and incomplete adenoma removal. Morcellation times were also reduced, potentially due to simplified attachment of fewer pieces, reducing bladder mucosa injury risk. The reduction in enucleation and morcellation time resulted in a significant decrease in the overall operative time for both the En-bloc and Two-lobe techniques compared to the Three-lobe technique. Despite the observed differences in these intraoperative parameters, the functional outcomes at the 3-month follow-up remained similar among the groups. These findings suggest that while the En-bloc and Two-lobe techniques may offer efficiency benefits and could be considered safe alternatives in ThuLEP procedures.

We noted a considerable decrease in laser enucleation time and laser energy administered in both the En-bloc and Two-lobe groups compared to the Three-lobe technique. However, there were no significant differences observed in laser enucleation time per gram or energy per gram, along with comparable rates of urgency-frequency symptoms and urge incontinence across all groups. While the En-bloc and Two-lobe techniques may offer efficiency benefits and could be considered safe alternatives in ThuLEP procedures, the reduction in laser enucleation time and energy delivered did not necessarily translate into improvements in postoperative storage symptom or other functional outcomes. In this scenario, the proficiency of the surgeon will be paramount in achieving favorable outcomes, as procedures are conducted with comparable energy release primarily through micromechanical action with the resector beak, irrespective of the surgical approach.

Nevertheless, we acknowledge that the En-bloc and Two-lobe techniques may be more challenging and require greater care, especially in large prostates, where it is easy to lose orientation. During the initial stages of the learning curve, it may be prudent to begin with the Three-lobe technique, despite requiring three identifications of the surgical plane. Enucleation of individual lobes in this technique greatly aids the surgeon and facilitates easier correction of any mistake.

This study is not without limitations. Although propensity score matching was utilized to mitigate confounding variables, residual confounding may persist due to unmeasured or unknown factors that were not included in the analysis. An additional limitation of this study is that the conclusions drawn regarding the efficiency of bilobar or en-bloc enucleation may not be universally applicable, as the familiarity and experience with different enucleation techniques, such as the trilobar approach, can vary significantly among surgeons at different tertiary centers. Future research should seek to address these limitations, potentially through larger-scale prospective studies involving diverse patient cohorts.

## Electronic supplementary material

Below is the link to the electronic supplementary material.

Supplementary Material 1

Supplementary Material 2

Supplementary Material 3

Supplementary Material 4

Supplementary Material 5

## Data Availability

Fabio Crocerossa had full access to all the data in the study and takes responsibility for the integrity of the data and the accuracy of the data analysis.
