# Supplementary material for: Refining surgical strategies in ThuLEP for BPH: a propensity score matched comparison of En-bloc, three lobes, and two lobes techniques
Source: World J Urol. 2024 Jul 22;42(1):431. doi: 10.1007/s00345-024-05136-5 (PMC11263241; doi:10.1007/s00345-024-05136-5)
Supplement: Supplementary file 1 — Supplementary Material 1 [file 345_2024_5136_MOESM1_ESM.docx]

***Supplementary Fig. 1*** *Post-hoc analysis of Laser Energy stratified by enucleation technique (En-bloc (n = 71) t,wo-lobe (n = 71), Three-lobe (n = 71). ET, enucleation time*
