# Supplementary material for: Refining surgical strategies in ThuLEP for BPH: a propensity score matched comparison of En-bloc, three lobes, and two lobes techniques
Source: World J Urol. 2024 Jul 22;42(1):431. doi: 10.1007/s00345-024-05136-5 (PMC11263241; doi:10.1007/s00345-024-05136-5)
Supplement: Supplementary file 2 — Supplementary Material 2 [file 345_2024_5136_MOESM2_ESM.docx]

***Supplementary Table 1*** *Preoperative characteristics of the study cohort stratified by enucleation technique (En-bloc (n=71), Two-lobe (n=71), Three-lobe (n=71). SD, Standard deviation; IPSS, International Prostate Symptom Score; Qmax, maximum urinary flow rate; PVR, postvoid residual urine volume; QoL, quality of life; 5-ARI, 5-alpha-reductase inhibitor; * Welch test; ** Kruskal-Wallis H test; ***Chi- squared test*

| **Variable** | **Treatment Group** | **Mean (SD)** | **Anova F** | ***p*** |
| --- | --- | --- | --- | --- |
| Age (years) | En-bloc | 69 (8.8) | 0.007 | *0.99* |
|  | Two-lobe | 68.8 (7.3) |  |  |
|  | Three-lobe | 68.9 (8.2) |  |  |
| IPSS Total (points) | En-bloc | 21.1 (8.2) | 2.245* | *0.11** |
|  | Two-lobe | 20.5 (6.8) |  |  |
|  | Three-lobe | 23.3 (9) |  |  |
| Qmax (ml/s) | En-bloc | 8.4 (3.2) | 1.481 | *0.23* |
|  | Two-lobe | 8.3 (2.9) |  |  |
|  | Three-lobe | 7.6 (3.2) |  |  |
| PVR (ml) | En-bloc | 142.6 (89) | 0.570 | *0.57* |
|  | Two-lobe | 129.3 (89.4) |  |  |
|  | Three-lobe | 143.9 (95.9) |  |  |
| Prostate Volume (ml) | En-bloc | 134.4 (59.1) | 0.246 | *0.78* |
|  | Two-lobe | 130.9 (63.2) |  |  |
|  | Three-lobe | 137.8 (53.6) |  |  |
| PSA (ng/ml) | En-bloc | 5.2 (2.3) | 1.696 | *0.19* |
|  | Two-lobe | 4.9 (2) |  |  |
|  | Three-lobe | 5.9 (3) |  |  |
| Hb (g/dl) | En-bloc | 12.9 (1.4) | 0.750 | *0.47* |
|  | Two-lobe | 12.6 (1.1) |  |  |
|  | Three-lobe | 12.8 (1.4) |  |  |
| ASA (points) | En-bloc | 2.7 (0.7) | 2.100** | *0.35*** |
|  | Two-lobe | 2.8 (0.8) |  |  |
|  | Three-lobe | 2.9 (0.8) |  |  |
| IPSS-QoL (points) | En-bloc | 3.1 (1.4) | 2.208** | *0.33*** |
|  | Two-lobe | 2.9 (1.1) |  |  |
|  | Three-lobe | 3.3 (1.4) |  |  |
| Indwelling Catheter |  | **NO (n,%)** | **YES (n,%)** |  |
|  | En-bloc | 55 (77.5%) | 16 (22.5%) | *0.93**** |
|  | Two-lobe | 54 (76.1%) | 17 (23.9%) |  |
|  | Three-lobe | 53 (74.6%) | 18 (25.4%) |  |
| 5-ARI Intake |  | **NO (n,%)** | **YES (n,%)** |  |
|  | En-bloc | 32 (45.1%) | 39 (54.9%) | *0.23**** |
|  | Two-lobe | 29 (40.8%) | 42 (59.2%) |  |
|  | Three-lobe | 39 (54.9%) | 32 (45.1%) |  |
