# Supplementary material for: Refining surgical strategies in ThuLEP for BPH: a propensity score matched comparison of En-bloc, three lobes, and two lobes techniques
Source: World J Urol. 2024 Jul 22;42(1):431. doi: 10.1007/s00345-024-05136-5 (PMC11263241; doi:10.1007/s00345-024-05136-5)
Supplement: Supplementary file 3 — Supplementary Material 3 [file 345_2024_5136_MOESM3_ESM.docx]

***Supplementary Table 2*** *Test of homogeneity of variances for preoperative variables. IPSS, International Prostate Symptom Score; Qmax, maximum urinary flow rate; PVR, postvoid residual urine volume*

|  |  | Levene Statistic | df1 | df2 | Sig. |
| --- | --- | --- | --- | --- | --- |
| Age | Based on Mean | 1.273 | 2 | 210 | 0.282 |
|  | Based on Median | 1.166 | 2 | 210 | 0.314 |
|  | Based on Median and with adjusted df | 1.166 | 2 | 205.862 | 0.314 |
|  | Based on trimmed mean | 1.262 | 2 | 210 | 0.285 |
| IPSS Total | Based on Mean | 4.349 | 2 | 210 | 0.014 |
|  | Based on Median | 2.238 | 2 | 210 | 0.109 |
|  | Based on Median and with adjusted df | 2.238 | 2 | 179.061 | 0.110 |
|  | Based on trimmed mean | 4.122 | 2 | 210 | 0.018 |
| Qmax | Based on Mean | 0.433 | 2 | 210 | 0.649 |
|  | Based on Median | 0.288 | 2 | 210 | 0.750 |
|  | Based on Median and with adjusted df | 0.288 | 2 | 202.969 | 0.750 |
|  | Based on trimmed mean | 0.420 | 2 | 210 | 0.658 |
| PVR | Based on Mean | 0.576 | 2 | 210 | 0.563 |
|  | Based on Median | 0.182 | 2 | 210 | 0.833 |
|  | Based on Median and with adjusted df | 0.182 | 2 | 206.127 | 0.833 |
|  | Based on trimmed mean | 0.465 | 2 | 210 | 0.629 |
| Prostate volume | Based on Mean | 0.556 | 2 | 210 | 0.574 |
|  | Based on Median | 0.179 | 2 | 210 | 0.836 |
|  | Based on Median and with adjusted df | 0.179 | 2 | 194.347 | 0.836 |
|  | Based on trimmed mean | 0.398 | 2 | 210 | 0.672 |
| PSA | Based on Mean | 1.970 | 2 | 210 | 0.142 |
|  | Based on Median | 1.116 | 2 | 210 | 0.330 |
|  | Based on Median and with adjusted df | 1.116 | 2 | 163.541 | 0.330 |
|  | Based on trimmed mean | 1.503 | 2 | 210 | 0.225 |
| Hb | Based on Mean | 2.373 | 2 | 210 | 0.096 |
|  | Based on Median | 2.063 | 2 | 210 | 0.130 |
|  | Based on Median and with adjusted df | 2.063 | 2 | 200.343 | 0.130 |
|  | Based on trimmed mean | 2.186 | 2 | 210 | 0.115 |
