# Supplementary material for: Refining surgical strategies in ThuLEP for BPH: a propensity score matched comparison of En-bloc, three lobes, and two lobes techniques
Source: World J Urol. 2024 Jul 22;42(1):431. doi: 10.1007/s00345-024-05136-5 (PMC11263241; doi:10.1007/s00345-024-05136-5)
Supplement: Supplementary file 4 — Supplementary Material 4 [file 345_2024_5136_MOESM4_ESM.docx]

***Supplementary Table 3*** *Multiple comparison test* *of intraoperative outcomes stratified by enucleation technique (En-bloc (n=71), Two-lobe (n=71), Three-lobe (n=71). ET, enucleation time; MT, morcellation time; LET, laser enucleation time; OT, operative time;* *The Tamhane's t2 test was applied for MT, MT per gram and OT comparison*

| Dependent Variable | (I) Group | (J) Group | Mean Difference (I-J) | Std. Error | *p* |  |
| --- | --- | --- | --- | --- | --- | --- |
|  |  |  |  |  |  |  |
| ET per gram (min/g) | En-bloc | Two-lobe | 0.00 | 0.03 | *0.996* |  |
|  |  | Three-lobe | -0.11 | 0.03 | ***<.001*** |  |
|  | Two-lobe | Three-lobe | -0.11 | 0.03 | ***0.002*** |  |
| ET (min) | En-bloc | Two-lobe | 4.10 | 2.85 | *0.661* |  |
|  |  | Three-lobe | -18.66 | 3.35 | ***<.001*** |  |
|  | Two-lobe | Three-lobe | -22.76 | 2.91 | ***<.001*** |  |
| MT per gram (min/g) | En-bloc | Two-lobe | -0.01 | 0.02 | *0.526* |  |
|  |  | Three-lobe | -0.12 | 0.02 | ***<.001*** |  |
|  | Two-lobe | Three-lobe | -0.11 | 0.02 | ***<.001*** |  |
| MT (min) | En-bloc | Two-lobe | -1.23 | 2.82 | *0.700* |  |
|  |  | Three-lobe | -16.47 | 2.89 | ***<.001*** |  |
|  | Two-lobe | Three-lobe | -15.24 | 3.43 | ***0.005*** |  |
| LET (min) | En-bloc | Two-lobe | 1.28 | 1.43 | *0.882* |  |
|  |  | Three-lobe | -7.06 | 1.43 | ***0.025*** |  |
|  | Two-lobe | Three-lobe | -8.34 | 1.43 | ***0.006*** |  |
| Energy (min) | En-bloc | Two-lobe | -12.70 | 7.13 | *0.219* |  |
|  |  | Three-lobe | -19.90 | 8.83 | *0.085* |  |
|  | Two-lobe | Three-lobe | -7.32 | 7.27 | *0.715* |  |
| OT per gram (min/g) | En-bloc | Two-lobe | -0.02 | 0.04 | *0.950* |  |
|  |  | Three-lobe | -0.23 | 0.03 | ***<.001*** |  |
|  | Two-lobe | Three-lobe | -0.22 | 0.04 | ***<.001*** |  |
| OT (min) | En-bloc | Two-lobe | 3.00 | 5.36 | *0.842* |  |
|  |  | Three-lobe | -34.89 | 5.36 | ***<.001*** |  |
|  | Two-lobe | Three-lobe | -37.89 | 5.36 | ***<.001*** |  |
