# Supplementary material for: Refining surgical strategies in ThuLEP for BPH: a propensity score matched comparison of En-bloc, three lobes, and two lobes techniques
Source: World J Urol. 2024 Jul 22;42(1):431. doi: 10.1007/s00345-024-05136-5 (PMC11263241; doi:10.1007/s00345-024-05136-5)
Supplement: Supplementary file 5 — Supplementary Material 5 [file 345_2024_5136_MOESM5_ESM.docx]

***Supplementary Table 4*** *Postoperative outcomes stratified by enucleation technique (En-bloc (n=71), Two-lobe (n=71), Three-lobe (n=71). LOS, Length of stay; Qmax, maximum urinary flow rate; PVR, postvoid residual urine volume; QoL, quality of life; * Kruskal-Wallis H test; **Chi- squared test*

| Variable | Treatment Group | N | Mean | Std. Deviation | Anova | |
| --- | --- | --- | --- | --- | --- | --- |
|  |  |  |  |  | F | *p* |
| Catheterization time (days) | En-bloc | 71 | 1.7 | 1.7 | 2.009 | *0.137* |
|  | Two-lobe | 71 | 1.8 | 2.0 |  |  |
|  | Three-lobe | 71 | 2.4 | 2.7 |  |  |
| LOS (days) | En-bloc | 71 | 1.8 | 1.5 | 0.707 | *0.494* |
|  | Two-lobe | 71 | 1.9 | 1.5 |  |  |
|  | Three-lobe | 71 | 2.2 | 2.3 |  |  |
| Postop Hb loss (g/dl) | En-bloc | 71 | 1.3 | 0.9 | 0.625 | *0.537* |
|  | Two-lobe | 71 | 1.4 | 1.1 |  |  |
|  | Three-lobe | 71 | 1.5 | 1.0 |  |  |
| Qmax change 3_mo (ml/s) | En-bloc | 71 | 8.9 | 4.8 | 1.242 | *0.291* |
|  | Two-lobe | 71 | 9.3 | 4.1 |  |  |
|  | Three-lobe | 71 | 10.1 | 4.7 |  |  |
| IPSS total change 3_mo (points) | En-bloc | 71 | -16.2 | 7.1 | 0.796 | *0.453* |
|  | Two-lobe | 71 | -15.8 | 6.3 |  |  |
|  | Three-lobe | 71 | -17.2 | 7.5 |  |  |
| PVR change 3_mo (ml) | En-bloc | 71 | -121.3 | 88.4 | 0.618 | *0.540* |
|  | Two-lobe | 71 | -108.0 | 92.7 |  |  |
|  | Three-lobe | 71 | -124.1 | 95.5 |  |  |
| PSA change 3_mo* (ng/ml) | En-bloc | 71 | 3.7 | 1.4 | 1.599 | *0.206* |
|  | Two-lobe | 71 | 3.5 | 1.2 |  |  |
|  | Three-lobe | 71 | 3.9 | 2.3 |  |  |
|  |  |  | Mean Rank | Kruskal-Wallis H | |  |
| IPSS QoL change 3_mo (points) | En-bloc | 71 | 100.9 | 2.4 | | *0.301** |
|  | Two-lobe | 71 | 104.6 |  |  |  |
|  | Three-lobe | 71 | 115.6 |  |  |  |
| Postoperative Minor Complications (Clavien≤2) |  | NO | % | YES | % |  |
|  | En-bloc | 60 | 34.9% | 11 | 26.8% | *0.563*** |
|  | Two-lobe | 55 | 32.0% | 16 | 39.0% |  |
|  | Three-lobe | 57 | 33.1% | 14 | 34.1% |  |
| Postoperative Major complications (Clavien>2) |  | NO | % | YES | % |  |
|  | En-bloc | 69 | 33.8% | 2 | 22.2% | *0.352*** |
|  | Two-lobe | 69 | 33.8% | 2 | 22.2% |  |
|  | Three-lobe | 66 | 32.4% | 5 | 55.6% |  |
| Postoperative Stress Incontinence |  | NO | % | YES | % |  |
|  | En-bloc | 70 | 33.5% | 1 | 25.0% | *0.775*** |
|  | Two-lobe | 70 | 33.5% | 1 | 25.0% |  |
|  | Three-lobe | 69 | 33.0% | 2 | 50.0% |  |
| Postoperative Urge Incontinence |  | NO | % | YES | % |  |
|  | En-bloc | 71 | 33.8% | 0 | 0.0% | *0.363*** |
|  | Two-lobe | 70 | 33.3% | 1 | 33.3% |  |
|  | Three-lobe | 69 | 32.9% | 2 | 66.7% |  |
